# Evaluation of the Little Rock Green Schoolyard initiative: a quasi-experimental study protocol

**DOI:** 10.1186/s12889-023-15891-6

**Published:** 2023-05-30

**Authors:** Matthew J. Barenie, Erin K. Howie, Kari A. Weber, Michael R. Thomsen

**Affiliations:** 1 Center for the Study of Obesity, Fay W. Boozman College of Public Health, University of Arkansas for Medical Sciences, Little Rock, AR USA; 2 Department of Health, Human Performance and Recreation, University of Arkansas, Fayetteville, AR USA; 3 Department of Epidemiology, Fay W. Boozman College of Public Health, University of Arkansas for Medical Sciences, Little Rock, AR USA; 4 Center for the Study of Obesity, Department of Health Policy and Management, Fay W. Boozman College of Public Health, University of Arkansas for Medical Sciences, 4301 W. Markham, Slot 820, Little Rock, AR 72205 USA

**Keywords:** Green space, Physical activity, Accelerometry, Childhood obesity, Green schoolyards, Child health, Quasi-experiment, Environmental strategies

## Abstract

**Background:**

Evidence suggests that access to green schoolyards may facilitate vigorous play and lead to increased physical activity, which could lead to improved academic outcomes and reduce excess childhood weight gain. Greener schoolyards can also provide additional outdoor amenities that help the community at large. The Little Rock Green Schoolyard Initiative, a program aiming to promote outdoor learning and play in two of the city’s community schools, provides a natural experiment to evaluate the role of such interventions. This article presents the protocols and study plans that will be used to evaluate this community-led initiative on several outcomes including physical activity, sleep quality, use of schoolgrounds, and perceptions of the school environment. Administrative datasets will be used to assess exposure to green schoolyard improvements on academic achievement, attendance, and disciplinary referrals during elementary school.

**Methods:**

Data will be gathered in two community schools where the green schoolyard improvements are taking place and in two demographically-matched comparison schools located elsewhere within the Little Rock School District. Data will be collected before, during, and after the green schoolyard improvements go into effect. Physical activity and sleep quality will be measured using actigraphy. Physical activity will also be assessed through direct playground observations during recess and outside of school hours. During the final year of the study, administrative data will be assembled and evaluated using difference-in-differences estimation and synthetic controls, two causal inference methods from the program evaluation literature.

**Discussion:**

The study is designed to provide new insights into the design, implementation, and evaluation of playgrounds among schoolchildren, especially those who are at risk of developing severe obesity during their elementary school years. The research herein will develop empirical data, elucidate potential mechanisms, and practical experience for future study, policymaking, and health services.

## Background

The Little Rock Green Schoolyard Initiative has an initial focus on two elementary schools. The initiative is supported with a training and technical assistance grant from the National League of Cities and the Children and Nature Network. The aim is to transform the grounds of these two schools into nature-filled greenspaces that are conducive to outdoor learning and provide better opportunities for physical activity during and after school hours. Site modifications that address drainage issues, renovations to activate existing greenspaces, improvements to fencing that facilitate easier access to green areas, and the addition of landscape plants to provide shade and improve air quality are among the planned green space improvements.

**Fig. 1:**
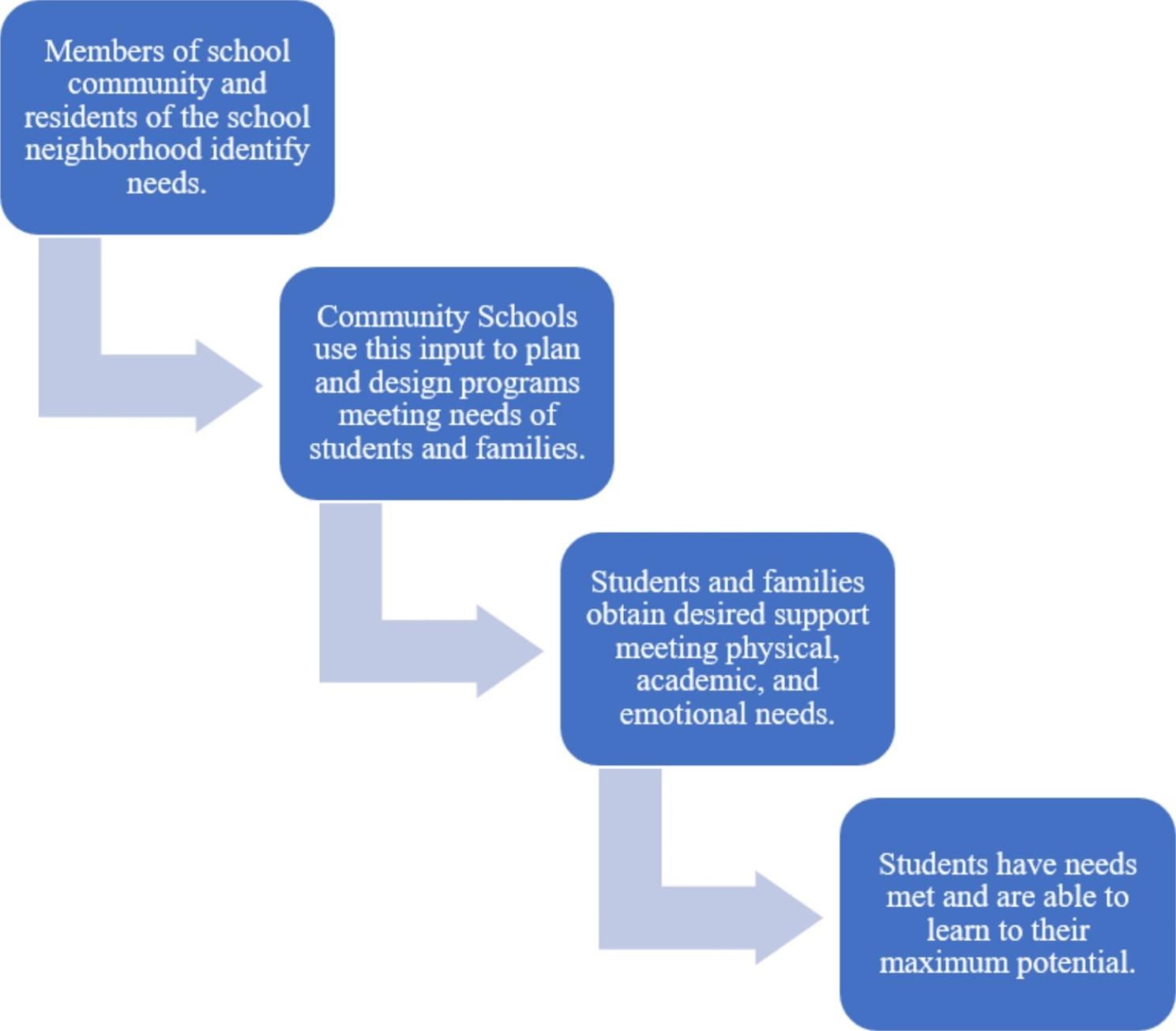
Overview of the community school model

The initiative is part of Little Rock Community Schools. Community schools engage members of the surrounding community to help ensure children have what they need to succeed (see Fig. 1). Using the community schools model, several critical needs were identified around school greenspace. These were: (1) opportunities for outdoor learning; (2) improved outdoor play experiences; and (3) safe, welcoming spaces for the community to connect with nature outside of school hours. The two schools initially targeted for green schoolyard improvements serve lower-income, predominately African American communities. Each school is centrally located in a walkable neighborhood, but residents in these neighborhoods have limited access to city parks within walking distance.

A growing literature on the health benefits of greener environments suggests that physical activity/play, air quality, sleep, and social behaviors are intertwined pathways that may protect against excess weight gain as well as promote psychological well-being and learning [1–12], hence the necessity of this work. Long term, programs like the Little Rock Green Schoolyard Initiative may improve health equity because the neighborhoods surrounding these schools are disproportionately affected by cardiometabolic conditions linked to inadequate physical activity and obesity.

**Fig. 2:**
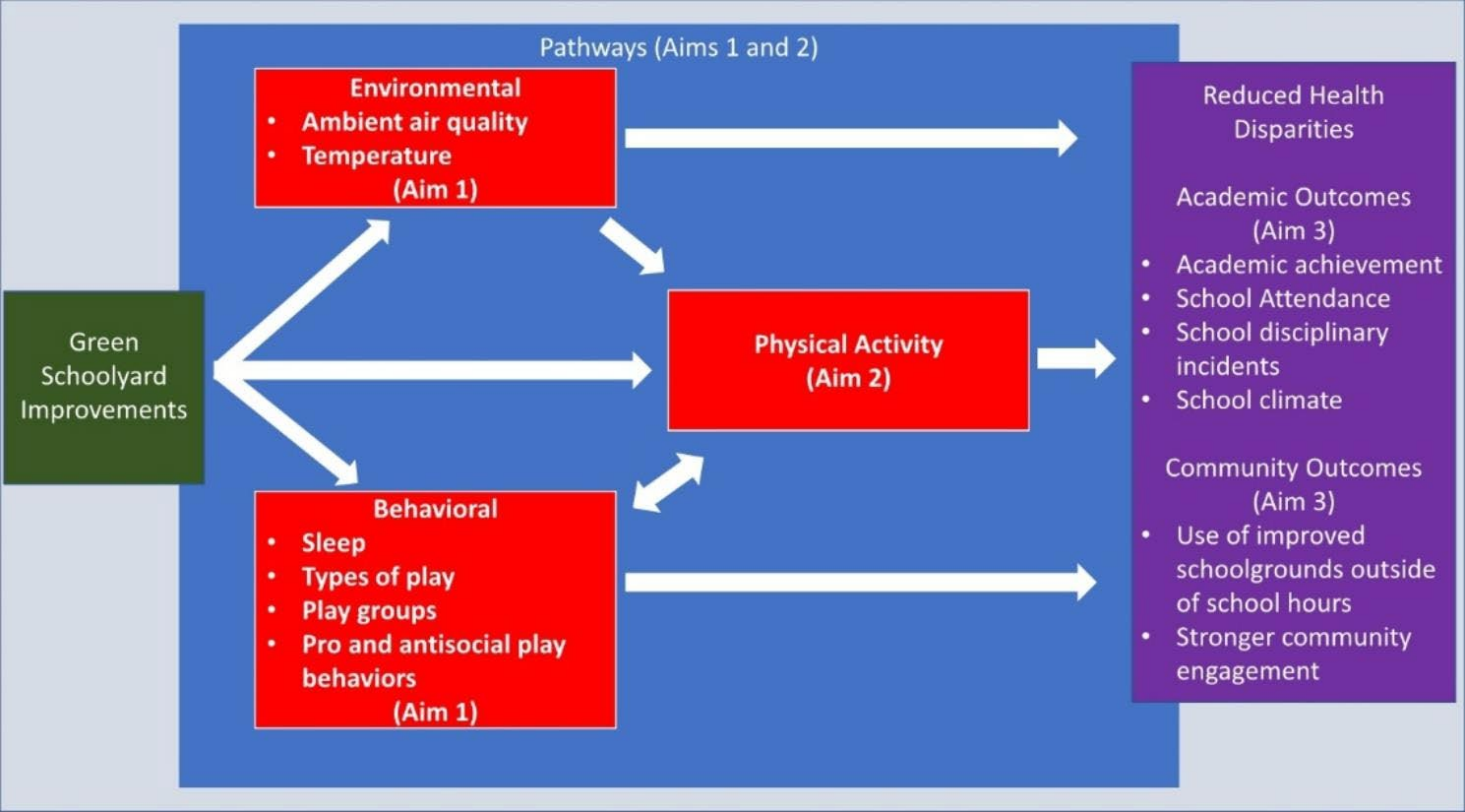
Conceptual model used to develop the aims of this study

The goal of this study is to take advantage of this community-led effort to understand the pathways through which green schoolyard improvements can improve health, academic outcomes, and community well-being (see Fig. 2). Specifically, the aims of the study are as follows:

1. Assess the impact of a community-led green schoolyard intervention on environmental and behavioral pathways to greater and more vigorous physical activity.
2. Determine the effect of greenspace improvements on physical activity among children in communities at high risk for cardiometabolic outcomes.
3. Identify the impact of greenspace improvements on academic and community-use outcomes of high importance to community- and school-level decision makers.

The study has an additional secondary aim, which is to characterize community- and school-level barriers that, if addressed, would amplify the impact of future school greenspace interventions.

## Methods

The study involves a pre-post-comparison design. Data will be collected on outcomes related to the pathways presented above in Fig. 2 before, during, and after the green schoolyard transformations from children in the program schools and in comparable (demographically-matched) non-program schools within the Little Rock School District. The study is designed to understand how these pathways work in combination and whether greener schoolyards influence patterns of play in ways that improve opportunities for physical activity among children who are at greater risk for developing severe obesity. Community- and school-level barriers to implementation of the green schoolyard intervention will also be identified to inform future initiatives. Table 1 summarizes data collection activities (described in more detail below) over the four-year study period.

**Table 1:** Summary of data collection measures by academic year (AY) and seasona^a^

	Baseline AY 1			Implementation AY 2			Post-implementation AY 3 and AY 4		
Data collection activity	Fa	Sp	Su	Fa	Sp	Su	Fa	Sp	Su
*Aim 1: Assess the impact of a community-led green schoolyard intervention on environmental and behavioral pathways to greater and more vigorous physical activity.*									
Accelerometry (sleep)^b^	X	X		X	X		X	X	
Social behaviors at recess (SOCARP)^c^	X	X		X	X		X	X	
Air quality/temperature	X	X	X	X	X	X	X	X	X
BMI measurement^d^	X	X		X	X		X	X	
*Aim 2: Determine the effect of greenspace improvements on physical activity among children in communities at high risk for cardiometabolic outcomes.*									
Accelerometry (physical activity)^b^	X	X		X	X		X	X	
Schoolground observations at recess (SOPLAY)	X	X		X	X		X	X	
Physical activity at recess (SOCARP)^c^	X	X		X	X		X	X	
BMI measurement^d^	X	X		X	X		X	X	
*Aim 3: Identify the impact of greenspace improvements on academic and community-use outcomes of high importance to community- and school-level decision makers.*									
School climate surveys	X	X		X	X		X	X	
After-hours schoolground observations (SOPLAY)	X	X	X	X	X	X	X	X	X
Passive monitoring of schoolground use	X	X	X	X	X	X	X	X	X
Retrospective analysis of administrative data on academic achievement, discipline, and attendance							X^e^	X^e^	X^e^
*Secondary Aim: Characterize community- and school-level barriers that, if addressed, would amplify the impact of future school greenspace interventions.*									
Teacher/staff interviews	X	X			X			X	

^a^ Su = summer, Fa = fall, and Sp = spring; SOCARP = System for Observing Children’s Activity and Relationships During Play, SOPLAY = System for Observing Play and Leisure Activity in Youth

^b^ Accelerometry for physical activity and sleep are listed separately by aim but are measured simultaneously

^c^ Collected during the same direct observation sessions

^d^ Measured when accelerometers are fitted, used to assess differences in playground behaviors and physical activity by weight status in Aims 1 and 2

^e^ Final post-implementation year (AY 4)

### Schoolground characteristics and use

Research assistants and the study team will systematically observe recess during the fall and spring of each academic year. Observations will be conducted using the System for Observing Play and Leisure Activity in Youth (SOPLAY) [13] and will assess activity, social context and environmental factors contributing to the quality of recess. With SOPLAY, members of the study team visually scan pre-determined target sections of the playground and record the number of persons engaged in various levels of physical activity at a given point in time [14–16]. Interobserver reliability will be regularly assessed. Observation days will be simultaneous between intervention and control school pairs and be conducted on a minimum of two school days within each fall and spring semester. Additional data collected on schoolground characteristics will be as follows:

- After-hours schoolyard observations using the SOPLAY protocol will also be conducted during weekend days and on randomly selected days during the summer, and non-academic term consistent with previous school-based observation protocols [16–18].
- Fine particulate matter (PM_2.5_) will be collected using sensors (Purple Air-II-SD, PurpleAir LIc, Utah, USA) that will be placed in the schoolyards of participating schools. The use of such monitors has been evaluated previously [19–24]. They are calibrated and highly correlated with each other, providing an expectation of validity when making comparisons of measures between and among the same Purple Air-II-SD monitors placed at different schools. Data will be collected on a continual basis and will be gathered periodically through storage on an SD card to provide PM_2.5_ data that can be linked temporally to the accelerometry data (described below) and to the playground observation data. These data will also be used to assess potential reductions in exposure to PM_2.5_ due to the greening of the school yards comparing pre- and post-implementation levels to those in the corresponding comparison schools.
- Temperature will also be tracked over time. Changes in land surface temperature and density of addition of greenness can be estimated over time using Landsat images from the US Geological Survey in Google Earth Engine and compared.

*Data collection involving schoolchildren*. School climate surveys, accelerometry, and recess observations will occur twice per year: once during the fall and again in the spring. At each data collection point, child/parent dyads will be recruited to participate in the following study activities.

d. *School climate surveys*: Consented/assented children in grades 3 to 5 will complete a short school climate survey covering the domains of engagement, safety and school environment using validated questions from the US Department of Education’s National Center on Safe and Supportive Learning Environments [25]. Participating children in kindergarten through grade 2 will not complete the climate survey because the survey questions are not designed for this younger age group.
e. *Accelerometer/Physical activity (and sleep) assessment*: Student total day, school day, and recess physical activity will be measured using GT9X accelerometers (ActiGraph, Pensacola, FL, USA) worn on the waist. Students will be asked to wear the devices on the hip for 7 days for 24 h per day except for water-based activities with parents reporting out-of-bed times and when the monitor is removed in accordance with existing protocols [26–30]. While consensus on processing 24-hour accelerometry data in children has yet to be established [31], waist placement is selected due to the established validity for estimating time spent in moderate-to-vigorous physical activity (MVPA) in children [32] and the ability to assess sleep duration in combination with reported sleep times [33]. For days not directly observed by research assistants, school start, and end times will be reported by the school, and recess times will be reported by classroom teachers daily with a sub-sample of recess periods observed by research assistants. Primary activity outcomes will be time spent in MVPA during the recess period, total school day and the total day in addition to total sleep time. Accelerometers will not be GPS capable due to the hesitancy of the target population to participate in research and parent unease with the location tracking of children.
f. *Biometric measures*: Children will be measured for height, weight, and waist circumference when the accelerometers are fitted. Height will be measured using a free-standing portable height rod (0044-0392-0 M, Detecto, Webb City, MO, USA). Weight will be measured using a personal scale (BF-689, Tanita, Arlington Heights, IL, USA) The measurement of waist circumference will occur via tape measure (BalanceFrom Body Tape Measure, Ontario, CA, USA) using standard World Health Organization procedures and internationally recommended cut points [34]. The fittings and measurements will take place in a private-setting within each of the schools (e.g., nurses office) and will follow current protocols for body mass index (BMI) measurement in Arkansas public schools [35].
  BMI will be calculated as (weight in pounds) ÷ (height in inches)^2^ × 703. Because recent findings caution against the use of BMI z-scores in analyses of samples with severely obese children [36–38], we will use %BMI_95_ defined as the ratio of BMI to the 95th percentile on the sex-specific BMI for age reference charts. Freedman and colleagues [38] showed that measures relative to the 95th percentile are more strongly correlated with other measures of adiposity than BMI z-scores and are correlated comparably to z-scores across the full distribution of children. Weight classifications used in the study will also be based on the Centers for Disease Control and Prevention sex- and age-specific BMI growth charts and will include underweight (BMI ≤ 5th percentile), normal weight (5th percentile ≤ BMI < 85th percentile), overweight (85th percentile ≤ BMI < 95th percentile), and obese (BMI ≥ 95th percentile). Those in the obese classification will further be assigned to class I obesity (100% ≤ %BMI_95_ < 120%), class II obesity (120% ≤ %BMI_95_ < 140%), and class III obesity (determined by the smaller of %BMI_95_ ≥ 140% of the 95th percentile or a BMI of 40) [39]. Finally, the assessment of waist circumference will enhance these BMI-based measures by allowing abdominal obesity to be measured. Abdominal obesity heightens the risk for insulin resistance and cardiovascular disease, among other detrimental physiological effects [40–42].

g. *Direct observations of target children during recess*: The SOPLAY protocol described above is used to measure the amount of different levels of physical activity in pre-determined target areas of the schoolground. A similar observation protocol, the System for Observing Children’s Activity and Relationships During Play (SOCARP) [43] will be modified and used to assess the activity of consented/assented children during recess. In our case, the target children are those participating in the accelerometry component of the study and for whom BMI and waist circumference will be measured. SOCARP is a validated protocol to measure physical activity levels on a single child in timed intervals [44]. The observers record information about activity types, social group size, and social interactions that can be used to assess occurrences of pro-social and anti-social behaviors before and after the greenspace enhancements [15, 45, 46]. We are also interested in whether these behaviors vary by gender and by weight status.
  In preparation for the recess observations and data collection, the study team developed R Shiny apps to collect the recess observation data. These apps take advantage of the open-source R software environment [47]. Separate apps are designed specifically for the playground observations during recess, observations outside of school hours, and individual recess observations of assented children. The apps are best used on a tablet computer with a local R installation or on mobile (iOS/iPadOS or Android) devices with one of the observers wearing a backpack server (a laptop in a backpack that is running R Shiny Server software and a battery-powered travel router). We anticipate releasing these apps under the open-source GNU General Public License (GPL) version 3 and making them available on GitHub after we have refined them in the field and have developed documentation to facilitate their use by other research groups.

*Administrative data on academic achievement, attendance, and disciplinary incidents.* During the final year of the study, we plan to obtain administrative educational data to evaluate the green schoolyard initiative in terms of academic achievement, attendance, and disciplinary referrals.

h. Academic achievement will be measured in terms of standardized test scores on math and English language arts (ELA) in 3rd through 5th grade. The actual tests used to assess children will vary during our study period causing scales of raw scores to differ over time depending on the test used. To address changes in testing, we will standardize the raw math and ELA scores by grade and year to obtain z-scores for use as the outcome.
i. Attendance will be measured as the fraction of days present at school to total school days.
j. Following earlier work using the discipline component of the Arkansas Department of Education (ADE) administrative data [48], we will measure total disciplinary infractions along with three subgroupings:
  i. Aggressive behavior infractions: Offenses such as fighting, bullying, student assault, or staff assault.
  ii. Misconduct infractions: Actions disruptive to the learning environment, such as disorderly conduct, insubordination, truancy, and vandalism.
  iii. Other infractions: Incidents related to contraband items and miscellaneous offenses including those that fall under the “other” category within the ADE data.

There is evidence of disparities in disciplinary enforcement. Schoolchildren from communities of color are charged with infractions at higher rates than non-Hispanic white children both nationally [49] and in Arkansas [48]. The difference in differences and synthetic control methods used in the statistical analyses described below are robust to this issue.

*Implementation measures.* The primary implementation constructs of fidelity, dose, reach, satisfaction, and context will be assessed from multiple sources [50]. Implementation monitoring will follow the Consolidated Framework for Implementation Research (CFIR) to systematically assess factors influencing implementation [51]. Qualitative and quantitative implementation data will be collected from multiple sources including record examination, observations, surveys, and interviews. These detailed assessments will help to understand the various influences on effectiveness, which is particularly important due to the distinctiveness of each site and the community influences beyond the control of the research project. Interviews will be conducted with key stakeholders including principals, teachers, and staff. Nurses and school counselors will be included to examine potential unintended consequences of the green schoolyards on injuries and student mental health. Interviews will be conducted using the CFIR Interview guide [52].

## Study population

### Inclusion criteria

The study population includes elementary school-aged children in participating schools, their parents, and educators (including teachers, administrators, and other school staff). The children will range from 4 years of age (Kindergarten) to 12 years of age (5th grade). Most children will be 5 to 11 years old, but the 4 to 12-year-old age range specified enables for children that enter kindergarten early or late or repeat a grade to qualify for this research. Parents and educators will vary in age.

### Exclusion criteria

Children, parents, and educators not attending or affiliated with one of the participating schools will not be eligible to participate.

Recruitment efforts will be coordinated and approved via the participating elementary schools and will occur multiple times per year during each of the four years of the study.

*Recruitment efforts*. IRB-approved recruitment flyers and signage will be used to recruit parent/child dyads. We will ask participating schools to include recruitment flyers along with other school and community interest information routinely being sent home to parents in student folders/backpacks and to distribute electronic versions of the flyer through routine electronic communications going to parents. We also plan to distribute flyers at school events attended by parents such as back-to-school nights, parent-teacher conferences, and PTA meetings. Signs will be placed in car-rider lines and near school entrances. IRB-approved invitations will be sent to educators with a request to participate in the CFIR interviews.

Our goal is to recruit up to 240 dyads (up to 10 per grade per school) at each of the planned data collection points. We will attempt to retain dyads for repeated observation over time but anticipate some degree of student turnover in these schools given that many families served by these schools face residential and job insecurity necessitating moves. Given the aims and scope of the study, we will not be retaining children who are no longer enrolled in the participating elementary schools due to a school transfer or to completion of the 5th grade.

## Data analysis

The data collection plan for physical activity outlined above will provide adequate power to detect meaningful effects. Using an alpha of 0.05 and power of 80%, we calculated the minimum detectable difference between two parallel groups in the primary outcome of MVPA during recess. In pilot data from several Arkansas elementary schools, the average time children spent in MVPA was 40.4% with a standard deviation of 18.7%. Assuming MVPA before the green schoolyards program, the minimum detectable difference would be 6.8% of recess time which is equal to or greater than previous interventions to increase recess physical activity [53]. Given the recruiting target N = 240) and a daily recess time of approximately 40 min as mandated by state legislation, we will have adequate power to detect anything greater than about a 3-minute difference in MVPA or 15 min per week.

Given the ability to collect baseline data, we can employ strong quasi-experimental designs to estimate the impact of the greener schoolyards on the pathway outcomes, the measurement of which are described above. We propose a difference-in-differences (DID) estimation to assess the program effects. DID is an established approach for causal inference in program and policy evaluations involving public health interventions [54]. We will assess whether our data meet the assumptions required to draw valid causal inferences from DID designs and will conduct balancing tests for similarity in pre-implementation characteristics between children in the program and comparison schools. If necessary, we will modify our design to estimate DID regressions from matched samples to improve pre-implementation balance [55]. Robust standard errors will be clustered by cohort within school.

Additional sensitivity analyses to better understand interactions between the pathway outcomes and to assess robustness of findings from the DID estimations will include mixed-effects models [56] to assess differences in the trajectory of the measured pathway outcomes between children in program and non-program schools. This will help determine whether the green schoolyard program differentially effects physical activity among children with an unhealthy weight status. Structural equation models to correct for correlated and unobserved effects in the errors [57] that could jointly determine physical activity and sleep are also appropriate for these data. Continual progress is being made on empirical methods to strengthen causal inferences in program evaluations [58].

The administrative data being used to assess academic achievement and discipline will contain elementary schools across the state allowing us to employ the synthetic control method. The use of synthetic controls [59–61] has been identified as one of the most important innovations for program and policy evaluation in recent years [58, 62]. In short, a “synthetic” control elementary school will be constructed to correspond to each program school. The construction of the synthetic control is a weighted average of schools that did not undergo greenspace renovations. The synthetic control approach will provide an estimate of the program effect for each of the two program schools. Inference about this effect is accomplished through a series of placebo tests wherein the true estimate of the program effect is compared to the distribution of program effects that results when the analysis is repeated for each non-program school and its synthetic control. It is also customary to test sensitivity of the estimated program effect to the inclusion/exclusion of control units from the set used by the algorithm to form the synthetic control. As noted above, we expect disparities in disciplinary instances due to differences in enforcement. These methods, DID, and synthetic controls are robust to this issue allowing us to estimate valid average program effects.

To analyze implementation, in contrast to deductive empirical studies which test an a priori hypothesis, analysis for this aim will take an inductive approach [63]. Investigator experience and systematic data collection will be integrated into a comprehensive case study analysis. Quantitative data will be summarized for each implementation construct, and thematic analysis will be utilized to analyze all qualitative data [64]. A primary concern of many critics of qualitative research is a lack of rigor [65, 66]. The credibility of the research will be strengthened through triangulation by including multiple data sources (i.e., administrators, teachers, students) and multiple methods (i.e., interviews and observations). These multiple sources will help to eliminate single source bias. Triangulation will serve as confirmation in addition to providing a holistic portrayal of the process of implementation [67].

## Discussion

The purpose of this research is to positively impact the lives and health of students, staff, and school communities by providing revamped and expanded green outdoor places for learning, playing, and engaging the community. A systematic review of experimental studies exploring the impact of schoolyard greening indicated favorable effects for physical activity and socioemotional health in children [68]. Not all greening/nature based interventions on childhood activity are found to be positive, some data show a decrease in moderate-vigorous physical activity when natural materials are added to a playground [69]. Similar to this natural experiment, previous work has shown the beneficial effects of green schoolyard renovations in low-income urban children by promoting higher levels of utilization, increased perception of safety, and more prosocial interactions [45]. The study herein helps address the limitations stated by Bohnert and colleagues by incorporating control/comparison schools and employing both observational and child level, via actigraphy, physical activity assessment methods [45].

Potential risk(s) of this research is the specific timing of program activities, changing administrations at the city and school level as well as potential adoption of greening programs at comparison schools is beyond the control of the study team. However, this study of the Little Rock Green Schoolyard Initiative provides an opportunity for a strong quasi-experimental design using demographically-matched comparison schools to answer important research questions and fill knowledge gaps in the literature on how greener schoolyards influence behaviors that reduce health disparities.

We anticipate that the Little Rock Green Schoolyard Initiative will be a new model for other schools to adopt and implement due to its potential to lessen health disparities, improve academic outcomes, and add a desirable area of recreation for the local community. Other regions with similar demographics can learn, modify, and benefit from this work. Future assessment of other demographics as well as specific mechanisms are necessary to evaluate the results of the Little Rock Green Schoolyard Initiative.

## Data Availability

Not applicable as this article does not present data.

## List of Abbreviations

BMI: Body mass index
DID: Difference-in-differences
MVPA: Moderate-to-vigorous physical activity
PM_2.5_: Fine particulate matter
SOPLAY: System for observing play and leisure activity in youth
SOCARP: System for observing children’s activity and relationships during play
